# Harnessing the Immune System Against Multiple Myeloma: Challenges and Opportunities

**DOI:** 10.3389/fonc.2020.606368

**Published:** 2021-01-27

**Authors:** Leona Yamamoto, Nicola Amodio, Annamaria Gulla, Kenneth Carl Anderson

**Affiliations:** ^1^Division of Hematologic Malignancy, Department of Medical Oncology, Jerome Lipper Multiple Myeloma Center, Dana-Farber Cancer Institute, Harvard Medical School, Boston, MA, United States; ^2^Department of Experimental and Clinical Medicine, Magna Graecia University of Catanzaro, Catanzaro, Italy

**Keywords:** myeloma, immunotherapy, microenvironment, immune system, challanges

## Abstract

Multiple myeloma (MM) is an incurable malignancy of plasma cells that grow within a permissive bone marrow microenvironment (BMM). The bone marrow milieu supports the malignant transformation both by promoting uncontrolled proliferation and resistance to cell death in MM cells, and by hampering the immune response against the tumor clone. Hence, it is expected that restoring host anti-MM immunity may provide therapeutic benefit for MM patients. Already several immunotherapeutic approaches have shown promising results in the clinical setting. In this review, we outline recent findings demonstrating the potential advantages of targeting the immunosuppressive bone marrow niche to restore effective anti-MM immunity. We discuss different approaches aiming to boost the effector function of T cells and/or exploit innate or adaptive immunity, and highlight novel therapeutic opportunities to increase the immunogenicity of the MM clone. We also discuss the main challenges that hamper the efficacy of immune-based approaches, including intrinsic resistance of MM cells to activated immune-effectors, as well as the protective role of the immune-suppressive and inflammatory bone marrow milieu. Targeting mechanisms to convert the immunologically “cold” to “hot” MM BMM may induce durable immune responses, which in turn may result in long-lasting clinical benefit, even in patient subgroups with high-risk features and poor survival.

## Introduction

Multiple myeloma (MM) is a plasma cell (PC) malignancy that accounts for approximately 1.5% of all cancers, and 10% of hematological malignancies (1). Abnormal proliferation of malignant PCs in the bone marrow (BM) in most cases leads to excessive secretion of immunoglobulin in the blood and urine, associated with organ dysfunction including hypercalcemia, renal dysfunction, anemia and/or bone disease (CRAB) (2). MM onset follows a multistep development process: tumor immune escape and accumulation of genomic aberrations in the malignant clone(s) drives the progression from precursor stages, namely monoclonal gammopathy of undetermined significance (MGUS) and smoldering multiple myeloma (SMM), to overt MM (1, 3). Current therapy consists of combination of novel agents with remarkable efficacy in MM. Specifically, combination proteasome inhibitor (PIs), immunomodulatory drugs (IMiDs), and dexamethasone used alone or integrated into high dose melphalan and autologous stem cell transplantation (ASCT) paradigm, increased rate, extent, and duration of response (4)..

Immunotherapy has recently demonstrated remarkable activity in many human solid tumors and is also transforming MM treatment as well. Recent approval of monoclonal antibodies (moAb) for the treatment of both newly diagnosed (NDMM) and relapsed/refractory (RRMM) MM patients highlights the fundamental role of therapies targeting the immunosuppressive microenvironment. The success of these approaches underlies the potential benefit of combination of immune- and targeted-therapies to overcome inter- and intra-patient heterogeneity characteristic of MM. In this review, we will discuss the role of the immunosuppressive BM microenvironment in MM, and outline novel immunotherapeutic approaches to effectively restore anti-MM immunity.

## Immune Dysfunction and Tumor Immune Evasion Mechanisms

BM-mediated immune dysfunction and tumor immune evasion represent the main challenges for immunotherapy in MM (5). However, although qualitative and/or quantitative alterations of cellular and non-cellular components of the BM *niche* in MM confer immunosuppression, they similarly represent ideal targets for novel therapeutics. Immune dysfunction not only confers MM cell growth and resistance to therapy, but also is associated with higher susceptibility to infections and impaired cellular immunity, evidenced by lack of a strong immune response to vaccinations (6–9). Alterations in accessory and immune cells in the BM including regulatory T cells, myeloid-derived suppressor cells (4, 10), Th17 cells, tumor-associated macrophages, mesenchymal stromal cells, and osteoclasts contribute to immune suppression and immune exhaustion (5, 11). Interaction of MM cells with plasmacytoid dendritic cells further promotes MM cell survival and therapy resistance, providing the rationale for targeting this interaction in novel therapeutic approaches (12, 13). Recent reports show a stepwise immune dysregulation in MM which occurs as early as in SMM stage, and the potential role of immune-based therapeutic interventions in premalignant precursor stages to delay or prevent progression to active MM in under active investigation in ongoing clinical trials (14–17).

During progression of disease, MM cells acquire the ability to evade the immune system and subvert cancer immunoediting, a dynamic process encompassing multiple aspects of tumor cell-immune system interactions (10, 18). Immunoediting, a process that is well described for solid tumors, shapes cancer cell immunogenicity in three phases: elimination, equilibrium, and escape. In the first phase, both innate and adaptive immunity recognize and eliminate early tumor cells (elimination). However, a state of dormancy next occurs in which a functional immune system maintains the survival of tumor cells under constant immune pressure (equilibrium). In this phase, resistant tumor cells acquire genetic and epigenetic alterations that eventually lead to escape the immune recognition, allowing for uncontrolled proliferation and clinical progression (escape) (19–21). A potential application of this model in MM identifies in the MGUS/SMM precursor stages a phase of immune equilibrium (22). In this context, marked heterogeneity of MM cells, along with constitutive and ongoing genomic instability, and modulations occurring in the composition of the BM *milieu* may underlie immune escape and disruption of the immune equilibrium during disease progression (22). Specifically, the strict and symbiotic interaction between MM cells and the BM microenvironment facilitate tumor immune escape *via* several mechanisms: immunosuppressive cells in the BM; disruption of antigen presentation by downregulating major histocompatibility complex and/or costimulatory molecules; loss or mutation of cancer-specific antigens; and upregulation of decoy receptors or complement inhibitory receptors (5, 23). Moreover, secretion of immunoregulatory soluble factors from both MM and BM microenvironment cells including transforming growth factor TGF-β, interleukin IL-10, IL-6, prostaglandin E2, and APRIL; as well as adhesion of MM cells to extracellular matrix proteins and accessory cells further promotes immune evasion and inhibition of apoptosis (5). Lastly, immune evasion also results from increased expression of immune checkpoints, i.e. PD-1/PD-L1, in T cells and MM cells, which has been associated with progression from precursor stages to clinically active MM, as well as with progression from NDMM to RRMM. As will be discussed later, clinically active agents blocking PD-1/PDL-1 axis have been associated with adverse events and are not approved for MM treatment; and ongoing studies are exploring the role of other potential immune checkpoint or agonist molecules including LAG 3 or TIGIT and OX40, respectively (24).

## Immunotherapy in MM

**Figure 1:**
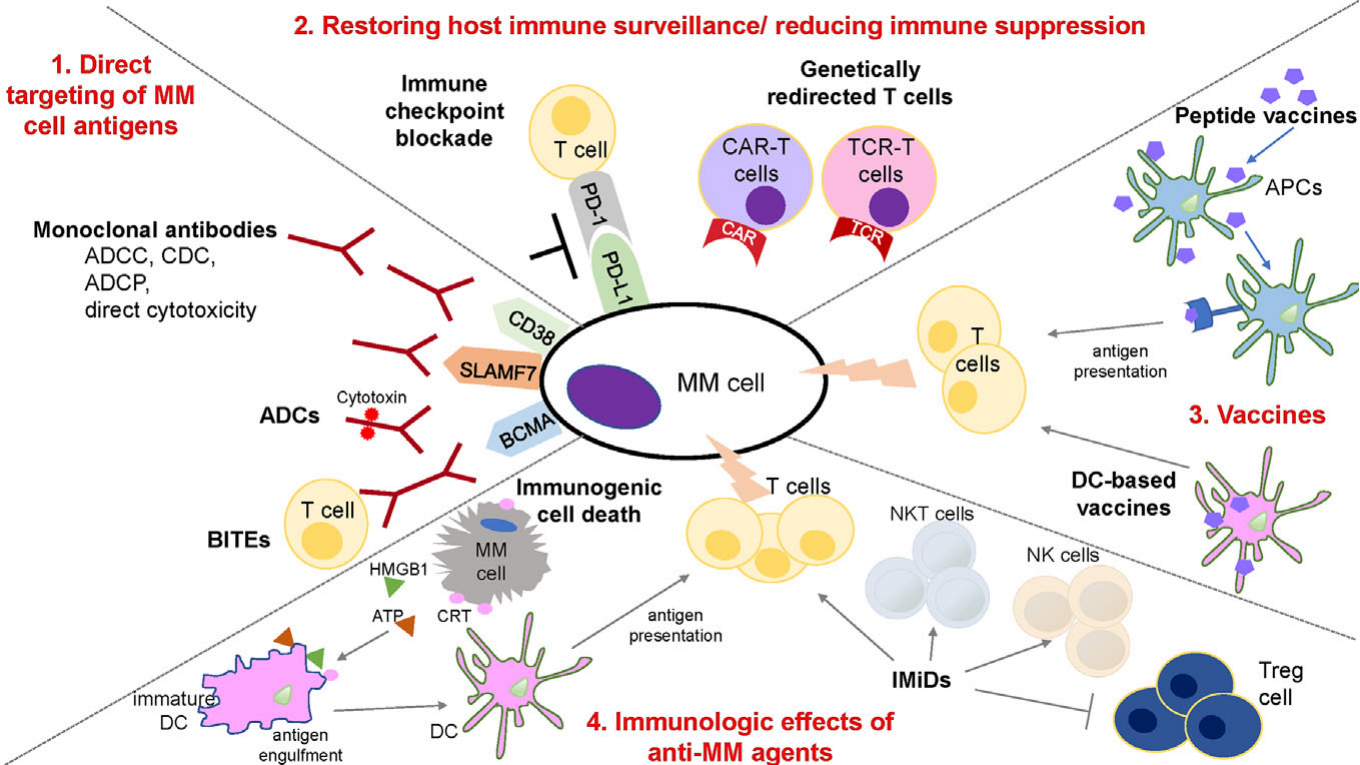
Schematic overview of immune therapies in MM. 1. Strategies for the direct targeting of MM cell antigens includes: (a) moAbs: anti-CD38 and -SLAMF7 antibodies induce ADCC, CDC, ADCP, and direct cytotoxic effect on MM cells; (b) ADCs: conjugation of moAbs and cytotoxic compounds provides direct and selective tumor killing; (c) BiTEs: dual interaction with surface antigen of tumor cells and the TCR complex enables T cell activation and tumor lysis of MM cells. 2. Restoration of host immune surveillance and decrease of immune suppression can be achieved by the blockade of immune-checkpoint, such as PD-1/PD-L1 axis, that are responsible for inactivation and loss of proliferative capacity of T cells; or by the use of genetically redirected T cells including CAR-T and TCR-T cells. CAR-T cells mediate MHC-unrestricted tumor cell killing *via* recognition of tumor antigen. TCR-T cells mediate MHC-restricted tumor cell killing by recognizing the intracellular antigen fragment presented by MHC molecules. 3. Peptide- or DC-based vaccination represents an additional strategy to increase a MM specific anti-tumor immunity. Peptide vaccines binds to restricted MHC molecule in APCs and after intracellular processing, peptide/MHC complex is transported to the cell surface for antigen presentation and activation of T cells. In DC-based vaccines, DCs are generated to present tumor associated antigens to T cells. 4. Anti-MM agents such as IMiDs and PIs may affect the immune compartment composition and increase anti-MM immune response. IMiDs increase and stimulate T, NK, and NKT cells, along with a decrease of immunosuppressive Treg cells. Novel reports also show the ability of anti-MM agents, such as bortezomib to induce immunogenic cell death (ICD) and stimulate an immune response against MM cells. Specifically, dying tumor cells expose specific damage associated molecular patterns (DAMPs) that induce the functional maturation of DCs, and the efficient presentation of tumor antigens to the T cells. *ADCC*, Antibody-dependent cellular cytotoxicity; *ADCP*, Antibody-dependent cellular phagocytosis; *CDC*, Complement-dependent cytotoxicity; *ADC*, Antibody drug conjugate; *BiTE*, Bi-specific T cell engager; *CAR*, Chimeric antigen receptor; *TCR*, T cell receptor; *APC*, Antigen presenting cell; *NK*, Natural Killer; *Treg*, regulatory T cells; *DC*, dendritic cell; *CRT*, Calreticulin.

The potential benefit of immunotherapeutic approaches in MM was first demonstrated by the curative effect achieved in some MM patients by the graft-*versus*-myeloma effect induced by allogeneic stem cell transplantation (23). Importantly, several moAbs and an immunotoxin have been FDA approved to treat MM and already improved patient outcome. Here, we review these therapies, as well as novel approaches under investigation in preclinical and clinical studies to further boost anti-MM immune response (**Figure 1**).

### Direct Targeting of MM Cell Tumor Antigens

#### Monoclonal Antibody Therapy

Monoclonal antibody (moAb) therapy is a successful strategy for both solid tumors and hematological malignancies; and recently has also transformed MM treatment (25). A key determinant for an optimal moAb efficacy is identification of a target uniquely or selectively and highly expressed by MM cells (26). To date, the targeting of two MM surface antigens, CD-38 and SLAMF7, has led to the Food and Drug Administration (FDA) approval of moAbs Daratumumab or Isatuximab and Elotuzumab, respectively, for MM treatment. Anti-MM immune effects of these moAbs include antibody-dependent cellular cytotoxicity (ADCC), antibody-dependent cellular phagocytosis (ADCP), complement activation, and direct effects on MM cells. Specifically, Elotuzumab is a humanized IgG1 monoclonal antibody that targets SLAMF7, which is highly expressed on PCs, natural killer (NK) cells, and activated monocytes (27). It induces ADCC while also activating NK cells and inhibiting MM cell adhesion to BMSCs (28, 29). Although no single agent activity has been reported in a phase I study (30), increased overall response (ORR) and median progression free survival (PFS) in the phase III Eloquent-2 trial provided the basis for its FDA approval in combination with lenalidomide and dexamethasone (Rd) in RRMM (31). More recently, the Eloquent-3 trial led to its approval in combination with pomalidomide and low dose dexamethasone (PomDex) in RRMM (32).

Daratumumab is an IgG1 kappa fully human moAb that targets CD38, which is highly expressed on malignant MM cells, but is also expressed on lymphoid and myeloid cells, hematopoietic progenitor cells, as well as non-hematopoietic tissues (18, 33). Anti-MM effects of daratumumab include: Fc fragment-dependent complement-dependent cytotoxicity (CDC), where the Fc fragment binds C1q, initiates complement cascade, and induces the formation of the membrane attack complex (MAC) that leads to MM cell lysis; ADCC, where Fc fragment binds an FcR-bearing effector cells such as natural killer (NK) cells, thus stimulating MM cytotoxicity; ADCP, where Fc fragment binds an Fc-bearing macrophage, thus stimulating MM cells phagocytosis; tumor cell apoptosis upon FcγR cross-linking (34–38); and immunomodulatory effects due to killing of CD-38-positive immune suppressor cells including regulatory T and B cells (Treg and Breg) and MDSCs (35), associated with an increase in T cell number, clonality, activation, and killing capacity due to higher levels of granzyme B (35). All these effects both induce MM cytotoxicity and increase host-anti MM response, associated with durable responses (**Table 1**). In the phase I/II GEN501 study and phase II SIRIUS study, Daratumumab monotherapy induced significant responses in heavily pretreated RRMM, leading to its FDA approval as monotherapy to treat RRMM in 2015 (39, 40). Combinations of daratumumab with Rd, PomDex, Bortezomib and dexamethasone (Vd), and carfilzomib dexamethasone are all FDA approved to treat RRMM; moreover, recent studies led to the FDA approval of Daratumumab in combination with Rd, or bortezomib, melphalan, and prednisolone (VMP), or bortezomib, thalidomide, and dexamethasone (VTd) to treat NDMM (41–46) due increased frequency and extent of durable responses (**Table 1**). A co-formulated product of daratumumab and recombinant human hyaluronidase PH20 suitable for subcutaneous administration has recently shown non-inferiority in the COLUMBA and PLEIADES trials as compared to intravenous infusion (47); and subcutaneous Daratumumab is now FDA approved, thus reducing infusion time and rate of infusion-related reactions.

**Table 1:** Daratumumab.

Treatment	Clinical trials	Patient population	ORR%	PFS	OS at 12 m	Date of approval
**D monotherapy**	**GEN501**	RRMM	36*	5.6 m	77%*	Nov 16, 2015
	**SIRIUS**	RRMM	29.2	3.7 m	64.8%	
	**POLLUX**	RRMM	92.9 *vs* 76.4	12 m: 83.2 *vs* 60.1%	92.1 *vs* 86.8%	Nov 21, 2016
**D-Rd vs Rd**						
	**MAIA**	NDMM transplant-ineligible	92.9 *vs* 81.3	30 m: 70.6 *vs* 55.6%		June 27, 2019
**D-Vd vs Vd**	**CASTOR**	RRMM	82.9 *vs* 63.2	12 m: 60.7 *vs* 26.9 %		Nov 21, 2016
**D plus PomDex**	**EQUULEUS**	RRMM	60	13.1 m: 8.8 m (median)	66% (median)	June 16, 2017
**D-VMP vs VMP**	**ALCYONE**	NDMM transplant-ineligible	90.9 *vs* 73.9	18 m: 71,6 *vs* 50.2%		May 7, 2018
**D-VTd vs VTd**	**CASSIOPEIA**	NDMM transplant-eligible	92.6 *vs* 89.9	18 m: 93 *vs* 85%		Sept 26, 2019
**D-RVd vs RVd**	**GRIFFIN**	NDMM transplant-eligible	99 *vs* 91.8	24 m: 95.8 *vs* 89.9 %		

*in the cohort of patients receiving 16 mg/kg dose.

NDMM, newly diagnosed MM patients; RRMM, relapsed refractory MM patients; D, daratumumab; R, lenalidomide; Pom, pomalidomide; V, bortezomib; M, melphalan; T, thalidomide; P, prednisone; d, dexamethasone; ORR, overall response rate; PFS, progression free survival; OS, overall survival; m, months.

Isatuximab (SAR650984) is another humanized IgG1 chimeric moAb that targets CD38. Its mechanisms of actions include CDC, ADCC, ADCP, and direct cytotoxicity without crosslinking of the Fc receptors of the antibody (48, 49). Moreover, both Daratumumab and Isatuximab may induce the depletion of CD38+ immune suppressor cells such as Tregs and Bregs (21, 50, 51). In the phase III ICARIA-MM study, isatuximab combined with PomDex showed superiority in terms of ORR (60.4 *vs* 35.3%) and median PFS (11.5 months *vs* 6.5 months) (52). This result led the FDA to approve isatuximab as a combination therapy with PomDex to treat RRMM in March 2020.

#### Antibody Drug Conjugate (ADC) and Bi-Specific Antibody (BiTEs)

Monoclonal antibodies have been recently used to develop Ab drug conjugates (ADC) and bispecific T cell engagers (BiTEs). ADCs are moAbs conjugated to cytotoxic compounds (such as auristatin) *via* synthetic linkers (53, 54). This conjugation provides both selective targeting and direct tumor killing, along with immune-mediated cytotoxicity (54–58). Several ADCs are under investigation in pre-clinical and clinical settings; among them, B cell maturation antigen (BCMA)-directed ADCs are showing promising effect due to the high and unique expression of BCMA on MM and late memory B cells, and to the oncogenic role of BCMA/APRIL pathway in the disease (56, 58, 59). Specifically, BCMA is a member of the tumor necrosis factor receptor superfamily (TNF) with high affinity for B cell activating factor (BAFF) and a proliferation-inducing ligand (APRIL). BCMA is essential for long-lived bone marrow PCs survival and regulates B cell differentiation into PCs (60, 61), thus representing an ideal target for MM therapy. Importantly, we have carried out preclinical studies of belantamab mafodotin, a BCMA-aurostatin immunotoxin, which recently received FDA approval in RRMM (58, 62). Balantamab mafodotin specifically blocks cell growth *via* G2/M arrest, induces caspase-dependent apoptosis, and ADCC (58). These multiple cytotoxic mechanisms enable potent and selective anti-MM activity.

BiTEs are bispecific antibodies which bind to specific tumor antigens on one side, and to the CD3 epsilon chain of the T-cell receptor complex on the other (63, 64). This dual interaction enables T cells engagement with tumor cells, which leads to T cell activation, cytolytic synapses, and tumor cell lysis (65, 66). In MM CD19, CD38, CD138, BCMA, GPRC5D, and Fc receptor-like 5 antigens have been tested (67–69), with early promising responses from BCMA BiTEs treatment in RRMM (66, 69). Novel bispecific antibodies are under clinical development and have shown encouraging data in preclinical studies. Specifically, AMG 701 holds an extended half-life *in vivo* and mediates T-cell dependent cellular cytotoxicity (TDCC) of BCMA positive MM cells and is currently under clinical investigation (NCT03287908). We have also shown that MM cytotoxicity can be augmented at lower effector: tumor cell ratios when low doses of AMG 701 are combined with lenalidomide or pomalidomide, suggesting a favorable therapeutic index (70). Although early results look promising and both ADC and Bites approaches have the advantage for “off the shelf” availability, longer follow-up in larger studies is needed to assess their clinical efficacy and toxicity.

### Restoring Host Immune Surveillance/Reducing Immune Suppression

#### Genetically Redirected T Cells

Cellular therapies represent an optimal strategy to restore host immune surveillance using either adoptive T-cell (ACT) or engineered T cell approaches (71–73). Expansion and activation of immunosuppressive T cells from the tumor microenvironment has shown best responses to date in solid tumors (72, 73). In MM, early experience showed that marrow-infiltrating lymphocytes (MILs) can be effectively used as a source of tumor specific T cells for ACT (74). Although these results are encouraging, ACT in MM has mainly utilized TCR and chimeric antigen receptor (CAR) T cell strategies, facilitated by recent progress in gene engineering technology (23). In the first approach, a TCR is cloned within patient T cells, thereby enabling specific recognition of patient’s tumor antigens in an MHC-dependent manner (71). Promising results were obtained in a phase I/II trial in which MM patients received high dose melphalan, ASCT, and two days later infusion of T cells engineered to express an affinity-enhanced TCR recognizing a naturally processed peptide shared by two cancer/testis antigens NY-ESO-1 and LAGE-1 (NY-ESOc259) (75, 76). Responses were noted in 80% patients with RRMM, and severe adverse events were not observed. Long-term persistence of NY-ESOc259 targeting T cells was detected and correlated with clinical activity against antigen-expressing MM cells (75). Importantly, CAR-T cell technology overcomes potential limitations of TCR-T cells by allowing recognition of unprocessed tumor antigen in an MHC-independent manner; however TCR, but not CAR-T, cells can also recognize intracellular proteins (23, 24). CARs are chimeric proteins that couple the constant region of a TCR and the tumor-associated antigen binding domain of a variable fragment of a moAb (23, 24, 77). Second-generation CAR-T cells also include costimulatory molecules, thus enhancing T cell activation and tumor killing by mimicking a physiological T cell response (23, 24, 77, 78). Patient’s T cells are engineered by means of electroporation, retroviral, or lentiviral transduction, expanded *ex vivo*, and then reinfused to the patient, leading to profound and rapid tumor killing (23, 24, 77). A major determinant of successful CAR-T therapy is the identification of the appropriate antigen, which is uniquely and highly expressed by MM cells, in order to avoid adverse events. Specifically, the main challenge of CAR-T therapy is the appropriate target selection, which is critical in the management of on-target, off-tumor toxicity to avoid excess cytokine release after target recognition on non-malignant cells (79). Similarly, the major on-target, on-tumor adverse events of CAR-T cells include cytokine release syndrome (CRS) characterized by fever, hypotension and/or renal failure, as well as neurotoxicity (80); and are mainly due to the CAR-T cell activation and expansion and uncontrolled cytokine release (79). Importantly, clinical experience has now shown that targeting interleukin-6 and use of dexamethasone can treat these toxicities (23, 24, 77, 81). Nonetheless, several strategies have been developed to overcome these toxicities and include either modifications of CAR-T cell activation kinetics to maintain activation and cytokine release under a controlled level, or approaches to restrict the recognition of normal cells by optimizing CAR-T/tumor cells interaction (79). Among several antigens including CD19, CD138 and SLAMF7, B-cell maturation antigen (BCMA) represents the most promising to date due to its high selective expression on normal plasma and MM cells (82, 83).

Several CAR-T products have been clinically evaluated in heavily pretreated (PIs-IMiDs-CD38 mAb) RRMM and demonstrated remarkable efficacy, including high rates of CR with minimal residual disease (MRD) negativity (77, 84). In the phase 1 study of bb2121, an anti-BCMA CAR-T, 85% of heavily pretreated RRMM patients had a clinical response lasting a median of 10.9 months without additional MM treatment (84). Encouraging results from the LEGEND-2 (NCT03090659) and CARTITUDE-1 (NCT03548207) clinical trials have been recently disclosed and show high overall response rate (ORR) with deep responses by using a different CAR-T product LCAR-B38M, also called JNJ 4528, which possesses a 4-1BB costimulatory domain and two BCMA-targeting domains (*Wang et al. ASH 2019; Madduri et al. ASH 2019*). Similarly, preliminary results from a phase 1 dose-escalating trial of a dual-target BM38 CAR recognizing both CD38 and BCMA are showing high ORR with a long duration of stringent CR and elimination of extramedullary lesions in RRMM patients (*Li et al., 2019 ASH*). However, relapse of disease occurs in most patients; and ongoing studies are evaluating mechanisms of CAR-T resistance in order to achieve durable responses. These include use of combination with immune approaches, treatment earlier in the disease course, and use of gamma secretase inhibitors to upregulate BCMA expression, among others.

#### Immune Checkpoint Blockade

Cancer cells may escape from T cell surveillance by altering the balance of costimulatory and coinhibitory molecular interactions. Stimulatory checkpoints and their ligands (CD27/CD70, CD40/CD40L, OX40/OX40L, GITR/GITL, CD137/CD137L, CD28/CD80 and CD86, ICOS/ICOSL) support T cell activation, whereas inhibitory checkpoints and their ligands (A2AR/adenosine, CTLA-4/CD80 and CD86, KIR/MHC class I, LAG3/MHC class II, PD-1/PD-L1 and PD-L2) lead to T cell suppression and induce their apoptosis (85). The PD-1/PD-L1 axis is the most studied pathway in MM. Programmed -death 1 (PD-1) receptor is a member of B7 family of costimulatory molecules and is expressed on antigen-activated and exhausted T, B, and NK cells (60, 86). PD-L1 expressing cells may evade T cell attack *via* several mechanisms, including induction of apoptosis, anergy or exhaustion of T cells, formation of a molecular shield to protect tumor cells from lysis, increased production of immunosuppressive IL-10, and stimulation of Treg cell-mediated suppression (87). Numerous studies have shown that PD-1 is overexpressed on CD4+ and CD8+ T cells of MM patients (88, 89), and that its expression is higher in patients with RRMM and MRD positive MM (90). Likewise, MM cells express PD-L1 at varying intensity (91–93), with a progressive increase in expression with progression from MGUS/SMM to NDMM to RRMM. Its oncogenic role in MM pathophysiology is also supported by the evidence that PD-L1 expressing MGUS or SMM show a rapid progression to symptomatic disease (94). MM cells-microenvironment interaction and secretion of proinflammatory cytokines (such as IL-6) promote PD-L1 upregulation on MM cell surface, which both inhibits T cell-mediated anti-MM immunity (94) and promotes MM cell survival by inducing reverse signaling to MM cells and activating the PI3K/AKT pathway (94). Although preclinical data have demonstrated the potential utility of PD-1/PD-L1 blockade in MM therapy, early clinical trials have been discouraging (95). Pembrolizumab immunotherapy did not show any activity in MM (96), and its combination with IMiDs, lenalidomide, or pomalidomide in RRMM patients was associated with immune-related toxicities and mortality in two phase III studies (KEYNOTE-183 and KEYNOTE-185), leading to a FDA clinical hold (24, 97). The precise mechanisms that lead to immune-related toxicities are still unknown (98); however, immune checkpoints are regulators of immunological homeostasis, and their functional disruption may unbalance immune tolerance that can lead to uncontrolled immune response (98, 99). Clinically, patients experience autoimmune-like/inflammatory reactions that can cause organ and tissue damages (98, 99). Although the mechanisms underlying the severity of such adverse events in some patients is yet to be elucidated, some reports suggest an association with underlying germline genetic factors or patient microbiota (98, 100). To date, the mechanisms underlying higher toxicity observed in MM patients as compared to other tumors are unknown. Pembrolizumab and nivolumab monotherapy in RRMM patients displayed similar safety profile as in other cancers (99); however, combination of pembrolizumab with IMiDs resulted in higher toxicity, with severe and unanticipated adverse events (99). Future analysis of combinations with other agents, patient selection, and timing of treatment initiation may allow for optimal therapeutical application of PD-1/PD-L1 axis blockade in MM. Recent studies suggest the potential role of other immune checkpoint or agonist proteins (i.e. LAG 3 or TIGIT and OX40, respectively) as MM therapeutic targets, alone and in combination with MM targeted and immune therapies (101). These novel studies, along with the identification of patients who may most benefit of immune checkpoint therapy, may allow for their future clinical use.

### Vaccine Strategies

Antigen-specific anti-tumor immunity can be primed by vaccination through several strategies including tumor cell-based vaccines (autologous or allogeneic), dendritic cell (DC)-based vaccines, protein/peptide-based vaccines, and genetic vaccines (DNA vaccines, RNA vaccine, viral based-vaccines) (102, 103). Among them, tumor cell-, protein/peptide-, and DC-based vaccines have been explored, although the main challenge to their efficacy has been represented by hallmark immunosuppression in MM (104, 105). GVAX (Aduro Biotech) is a vaccine platform that uses tumor cells which are genetically engineered to produce granulocyte-macrophage colony-stimulating factor GM-CSF (106), which can recruit and activate DCs and other APCs (107). This vaccine is now being tested in combination with lenalidomide in patients with CR and near CR (NCT03376477). Several antigens that are broadly expressed in MM cells, such as MAGE-A3, WT-1, SLAMF7, CD138, and XBP-1 have been examined alone or in combination in the context of peptide/protein-based vaccines (108–110). Although the clinical efficacy seems modest, new approaches using a multipeptide-based vaccine induced an effective and durable memory multipeptide-specific cytotoxic T lymphocytes response in patients with SMM, suggesting its potential utility to delay disease progression (111). Similarly, a novel engineered heteroclitic BCMA peptide can induce BCMA-specific memory immunity, providing the rationale for its clinical evaluation (112). Additionally, previous reports have also explored the idiotype (Id) vaccination in which early-stage MM patients are immunized with autologous tumor-derived clone-specific immunoglobulin, both as a peptide vaccine and a DNA vaccine (113–115). Id specific immunological responses were reported, although clinical responses were infrequent.

DC-based vaccines are also under investigation in MM. Two approaches can be used to augment presentation of tumor antigens in DC: to chemically fuse patient DCs with autologous MM cells (MM cells/DCs fusion vaccine); or to load DCs with tumor antigens in the form of peptides, proteins, tumor lysates, and mRNAs (106, 116). In the first phase I clinical trial, 16 patients were treated with MM cell/DC fusion vaccine with GM-CSF as an adjuvant. The majority of patients (11 of 15) showed disease stabilization, associated with expansion of circulating CD4+ and CD8+ lymphocytes reactive against autologous MM cells (116). In the phase II trial, patients received vaccination post-ASCT. In this setting, vaccination induced effective anti-MM immunity and increases the depth of response, with 78% of the patients showing CR/VGPR (117). Moreover, 24% patients converted from PR to CR/near CR after vaccination at more than 3 months post-ASCT, suggesting a vaccine-mediated effect targeting residual disease (117). To confirm these results, phase II trial in the same setting (NCT02728102) is ongoing. Another DC-based vaccine approach in which Langerhans-type DCs (LCs) are electroporated with mRNAs encoding MM antigens CT7, MAGE-A3, and WT-1 is now being evaluated in the post-ASCT setting in phase I clinical trial (NCT01995708). This trial is based on preclinical data showing that LCs induce a more potent T cell response than monocyte-derived DCs, and that the electroporation of mRNA stimulates their maturation and activation (118, 119).

Of note, due to the lack of an optimal response after vaccination in MM patients to date, an ongoing trial (NCT02728102) is exploring whether combination with IMiDs may increase a clinically significant immune response.

### Immunologic Effects of Anti-MM Agents

Several studies have focused on the effects of anti-MM agents on cellular and non-cellular components of the bone marrow microenvironment, including immune cells. Immunomodulatory agents (IMiDs) thalidomide and its more potent derivatives lenalidomide and pomalidomide represent the best example of drugs with both direct cytotoxic activity on MM cells and immunomodulatory effects (24). Thalidomide is approved to treat both NDMM and RRMM in combination with bortezomib and dexamethasone (VTd), and the recent phase III CASSIOPEIA study granted its FDA approval in combination with daratumumab (D-VTd) in NDMM (46). However, occurrence of peripheral neuropathy and introduction of lenalidomide and pomalidomide in the MM armamentarium, has progressively decreased its clinical use. Lenalidomide is used in combination with bortezomib and dexamethasone (RVd) to treat transplant-eligible NDMM (120, 121); a dose modification (RVd-lite) has also shown a favorable therapeutic index in transplant-ineligible NDMM patients (121, 122). Lenalidomide is also FDA approved post-ASCT as maintenance therapy as it prolongs both PFS and OS (123); in RRMM setting, it is FDA approved in combination with daratumumab (41), elotuzumab (31), ixazomib (124), and carfilzomib (125). Pomalidomide is FDA approved to treat RRMM patients in combination with dexamethasone along with elotuzumab, bortezomib, daratumumab, and isatuximab (32, 44, 126), and its role in this setting has become more evident due to the broad use of lenalidomide in both NDMM and in maintenance and the development of lenalidomide resistance leading to relapse. Elegant mechanistic studies identified cereblon (CRBN) as a major target for IMiDs in MM cells, and showed that they induce growth arrest and caspase-8–mediated apoptosis, associated with CRBN-dependent degradation of Ikaros (IKZF1) and aiolos (IKZF3) transcription factors, followed by IRF4 downregulation (24, 127, 128). IMiDs treatment impacts the MM cell/microenvironment interaction by decreasing cell surface expression of adhesion molecules, modulating cytokine and growth factor secretion, and inhibiting angiogenesis (24). Immunomodulatory effects include activation of cytotoxic CD8+ T, NK, and NKT cells, along with a decrease of Treg (24). CRBN targeting is also responsible for the immune effects of IMiDs, as degradation of IKZF1/3 in T cells increases IL-2 secretion (129) and NK and NKT cell cytotoxicity (24). More recently, it has been shown that IMiDs can enhance NK and T cell cytotoxicity by triggering granzyme-B *via* either CRBN or ZAP-70 dependent mechanisms, thus providing the rationale for novel therapeutics to activate ZAP-70 in MM (130). Recent studies are also suggesting different mechanisms of action between pomalidomide and lenalidomide *in vivo*, consistent with clinical responses observed in patients with lenalidomide RRMM (131, 132).

Importantly, the identification of the mechanism of action of IMiDs has informed the development of a new class of drugs, CELMoD agents which are higher affinity CRBN E3-ligase modulators (24). Among them, Iberdomide and CC-92480 have shown significant preclinical activity and are currently under investigation in the RRMM clinical setting (133, 134).

Proteasome Inhibitors (PI) bortezomib, carfilzomib, and ixazomib represent the backbone of MM therapy in both NDMM and RRMM. MM cells are highly dependent on proteasome activity due to their high turnover of abnormal immunoglobulins (24, 135). Although PIs exert a primarily cytotoxic effect on MM cells, the biological outcome of proteasome inhibition also targets the MM microenvironment. PI treatment can disrupt MM cell/bone marrow cell adhesion by decreasing the expression of adhesion molecules, inhibit angiogenesis by modulating secretion of several cytokines, and modify osteoclast activity and bone turnover (136). However, the effect of this class of agents on the immune cells is still largely unknown, with preclinical data suggesting an immunosuppressive role (137). Interestingly, a more recent elegant study has instead shown an immunogenic potential role of bortezomib in eliciting an anti-MM immune response *in vitro* (138). Moreover, our recent studies using both syngeneic *in vivo* MM model and MM patient samples show that bortezomib treatment triggers immunogenic MM cell death, which in turn primes an effective anti-MM immune response and disease control *in vivo* models and in patients (*Gulla et al., ASH 2019*). A deep understanding of the immunomodulatory role of PIs will be instrumental to inform their clinical use in combination with immune therapies.

As shown in several types of cancers, histone deacetylase (HDAC) inhibitors are powerful epigenetic regulators with a wide range of effects, including immune modulation (139). For example, recent evidence has shown that an HDAC6 specific inhibitor ACY241 exerts its anti-MM activity, at least in part, by enhancing anti-tumor response of antigen-specific central memory cytotoxic T lymphocytes against MM (140). Future studies will better inform the therapeutic use of HDAC inhibitors as immune regulators in this disease.

## Discussions

Increasing knowledge of MM pathobiology and immune microenvironment dysfunction, along with the introduction of PIs and IMiDs-based regimens, has already transformed patient outcome in MM patients. The advent of immunotherapy in MM has already shown remarkable effects in terms of extent and frequency of response. Moreover, immune-based approaches, alone and in combination, have the potential to overcome not only immune dysfunction, but also constitutive and ongoing genomic heterogeneity of MM cells, and thereby improve patient long-term control of disease. However, several challenges remain for effective translation of novel immune strategies into clinical practice, as well as for optimal clinical use of drugs including moAbs that are already incorporated in the treatment regimens (141). Clinical challenges are associated with moAbs use, including infusion reactions, infection risk and blood typing interference that causes positive indirect Coombs test occurring with Daratumumab treatment. Moreover no clear data are available that identify alternative combinations besides their incorporation in triplet regimens, or the characteristic of the patient population in which Daratumumab may be preferably used as single drug (141).

Similarly, resistance to immune approaches hamper their long-term efficacy and may develop due to the loss of target antigen or immune suppression. To address this concern, novel approaches targeting multiple antigens are under investigation (24). Along with loss of surface expression of target protein, antigen in soluble form may potentially interfere with immune-targeted strategies. For example, soluble BCMA, which is cleaved by γ-secretase, inhibits CART cell recognition of surface BCMA (142, 143). High levels of soluble BCMA are present in RRMM, and a clinical trial is testing the combination of anti-BCMA CAR-T therapy with γ-secretase inhibitor to block BCMA cleavage from the MM cell surface (NCT03502577). Although CAR-T therapies represent a very promising strategy, several challenges intrinsic to the technology may limit their efficacy; and ongoing efforts are optimizing their design to avoid antigen-independent tonic signaling, and to increase their expansion and persistence *in vivo*, by enriching for early memory T cell phenotype and/or intensifying lymphodepletion to promote CAR-T persistence (77). Moreover, the optimal timing for immune intervention during MM the disease course remains undefined. Early immune-based intervention in high-risk SMM patients to avoid development of active MM is promising strategy, but must be balanced against adverse events and therapeutic index. Finally, correlative studies using MM patient samples will delineate mechanisms of action and resistance and thereby inform clinical application of immune therapies in combination with other anti-MM agents. Restoring host anti-MM immunity along with MRD negativity will be required for the long-term control of disease and its potential cure.

## Author Contributions

AG and KA conceived the review. LY and AG collected the literature. LY, AG, and NA drafted the manuscript and prepared the table and figure. KA provided critical comments on the manuscript—review and editing. All authors contributed to the article and approved the submitted version.

## Funding

This work is supported by NIH/NCI grants SPORE-P50CA100707 (KA), R01-CA050947 (KA), R01CA207237 (KA), P01CA155258 (KA), and R01-CA178264 (KA); the Sheldon and Miriam Medical Research Foundation (KA), and Italian Ministry of Health GR-2016-02361523 (NA). KA is an American Cancer Society Clinical Research Professor. AG is a Fellow of The Leukemia & Lymphoma Society and a Scholar of the American Society of Hematology (ASH).

## Conflict of Interest

KA serves on advisory boards to Celgene, Millennium, Janssen, Sanofi, Bristol Myers Squibb, Gilead, Precision Biosciences, and Tolero and is a Scientific Founder of OncoPep andC4 Therapeutics.

The remaining authors declare that the research was conducted in the absence of any commercial or financial relationships that could be construed as a potential conflict of interest.

The reviewers SO and MC declared a past co-authorship with one of the authors, respectively, KA and NA to the handling editor.

## References

[1] Kumar SK Rajkumar V Kyle RA van Duin M Sonneveld P Mateos MV Multiple myeloma. Nat Rev Dis Primers (2017) 3:17046. 10.1038/nrdp.2017.46 28726797

[2] Palumbo A Anderson K Multiple myeloma. N Engl J Med (2011) 364(11):1046–60. 10.1056/NEJMra1011442 21410373

[3] Landgren O Kyle RA Pfeiffer RM Katzmann JA Caporaso NE Hayes RB Monoclonal gammopathy of undetermined significance (MGUS) consistently precedes multiple myeloma: a prospective study. Blood (2009) 113(22):5412–7. 10.1182/blood-2008-12-194241 PMC2689042 19179464

[4] Guang MHZ McCann A Bianchi G Zhang L Dowling P Bazou D Overcoming multiple myeloma drug resistance in the era of cancer ‘omics’. Leuk Lymphoma (2018) 59(3):542–61. 10.1080/10428194.2017.1337115 PMC6152877 28610537

[5] Holthof LC Mutis T Challenges for Immunotherapy in Multiple Myeloma: Bone Marrow Microenvironment-Mediated Immune Suppression and Immune Resistance. Cancers (Basel) (2020) 12(4). 10.3390/cancers12040988 PMC7226482 32316450

[6] Blimark C Holmberg E Mellqvist UH Landgren O Bjorkholm M Hultcrantz M Multiple myeloma and infections: a population-based study on 9253 multiple myeloma patients. Haematologica (2015) 100(1):107–13. 10.3324/haematol.2014.107714 PMC4281323 25344526

[7] Kristinsson SY Tang M Pfeiffer RM Bjorkholm M Goldin LR Blimark C Monoclonal gammopathy of undetermined significance and risk of infections: a population-based study. Haematologica (2012) 97(6):854–8. 10.3324/haematol.2011.054015 PMC3366650 22180421

[8] Robertson JD Nagesh K Jowitt SN Dougal M Anderson H Mutton K Immunogenicity of vaccination against influenza, Streptococcus pneumoniae and Haemophilus influenzae type B in patients with multiple myeloma. Br J Cancer (2000) 82(7):1261–5. 10.1054/bjoc.1999.1088 PMC2374477 10755398

[9] Ljungman P Nahi H Linde A Vaccination of patients with haematological malignancies with one or two doses of influenza vaccine: a randomised study. Br J Haematol (2005) 130(1):96–8. 10.1111/j.1365-2141.2005.05582.x 15982350

[10] Gorgun GT Whitehill G Anderson JL Hideshima T Maguire C Laubach J Tumor-promoting immune-suppressive myeloid-derived suppressor cells in the multiple myeloma microenvironment in humans. Blood (2013) 121(15):2975–87. 10.1182/blood-2012-08-448548 PMC3624943 23321256

[11] Tamura H Immunopathogenesis and immunotherapy of multiple myeloma. Int J Hematol (2018) 107(3):278–85. 10.1007/s12185-018-2405-7 29368256

[12] Chauhan D Singh AV Brahmandam M Carrasco R Bandi M Hideshima T Functional interaction of plasmacytoid dendritic cells with multiple myeloma cells: a therapeutic target. Cancer Cell (2009) 16(4):309–23. 10.1016/j.ccr.2009.08.019 PMC2762396 19800576

[13] Ray A Song Y Chauhan D Anderson KC Blockade of ubiquitin receptor Rpn13 in plasmacytoid dendritic cells triggers anti-myeloma immunity. Blood Cancer J (2019) 9(8):64. 10.1038/s41408-019-0224-6 31406111 PMC6690908

[14] Das R Strowig T Verma R Koduru S Hafemann A Hopf S Microenvironment-dependent growth of preneoplastic and malignant plasma cells in humanized mice. Nat Med (2016) 22(11):1351–7. 10.1038/nm.4202 PMC5101153 27723723

[15] Glavey SV Naba A Manier S Clauser K Tahri S Park J Proteomic characterization of human multiple myeloma bone marrow extracellular matrix. Leukemia (2017) 31(11):2426–34. 10.1038/leu.2017.102 28344315

[16] Paiva B Mateos MV Sanchez-Abarca LI Puig N Vidriales MB Lopez-Corral L Immune status of high-risk smoldering multiple myeloma patients and its therapeutic modulation under LenDex: a longitudinal analysis. Blood (2016) 127(9):1151–62. 10.1182/blood-2015-10-662320 26668134

[17] Mateos MV Hernandez MT Giraldo P de la Rubia J de Arriba F Lopez Corral L Lenalidomide plus dexamethasone for high-risk smoldering multiple myeloma. N Engl J Med (2013) 369(5):438–47. 10.1056/NEJMoa1300439 23902483

[18] Malavasi F Deaglio S Funaro A Ferrero E Horenstein AL Ortolan E Evolution and function of the ADP ribosyl cyclase/CD38 gene family in physiology and pathology. Physiol Rev (2008) 88(3):841–86. 10.1152/physrev.00035.2007 18626062

[19] Dunn GP Bruce AT Ikeda H Old LJ Schreiber RD Cancer immunoediting: from immunosurveillance to tumor escape. Nat Immunol (2002) 3(11):991–8. 10.1038/ni1102-991 12407406

[20] Mittal D Gubin MM Schreiber RD Smyth MJ New insights into cancer immunoediting and its three component phases–elimination, equilibrium and escape. Curr Opin Immunol (2014) 27:16–25. 10.1016/j.coi.2014.01.004 24531241 PMC4388310

[21] Ho M Goh CY Patel A Staunton S O’Connor R Godeau M Role of the Bone Marrow Milieu in Multiple Myeloma Progression and Therapeutic Resistance. Clin Lymphoma Myeloma Leuk (2020) 20(10):e752–68. 10.1016/j.clml.2020.05.026 32651110

[22] Minnie SA Hill GR Immunotherapy of multiple myeloma. J Clin Invest (2020) 130(4):1565–75. 10.1172/JCI129205 PMC7108923 32149732

[23] Rodriguez-Otero P Paiva B Engelhardt M Prosper F San Miguel JF Is immunotherapy here to stay in multiple myeloma? Haematologica (2017) 102(3):423–32. 10.3324/haematol.2016.152504 PMC5394971 28082344

[24] Gulla A Anderson KC Multiple myeloma: the (r)evolution of current therapy and a glance into future. Haematologica (2020) 105(10):2358–67. 10.3324/haematol.2020.247015 PMC7556665 33054076

[25] Scott AM Allison JP Wolchok JD Monoclonal antibodies in cancer therapy. Cancer Immun (2012) 12:14. 22896759 PMC3380347

[26] Scott AM Wolchok JD Old LJ Antibody therapy of cancer. Nat Rev Cancer (2012) 12(4):278–87. 10.1038/nrc3236 22437872

[27] Hsi ED Steinle R Balasa B Szmania S Draksharapu A Shum BP CS1, a potential new therapeutic antibody target for the treatment of multiple myeloma. Clin Cancer Res (2008) 14(9):2775–84. 10.1158/1078-0432.CCR-07-4246 PMC4433038 18451245

[28] Tai YT Dillon M Song W Leiba M Li XF Burger P Anti-CS1 humanized monoclonal antibody HuLuc63 inhibits myeloma cell adhesion and induces antibody-dependent cellular cytotoxicity in the bone marrow milieu. Blood (2008) 112(4):1329–37. 10.1182/blood-2007-08-107292 PMC2515112 17906076

[29] Collins SM Bakan CE Swartzel GD Hofmeister CC Efebera YA Kwon H Elotuzumab directly enhances NK cell cytotoxicity against myeloma via CS1 ligation: evidence for augmented NK cell function complementing ADCC. Cancer Immunol Immunother (2013) 62(12):1841–9. 10.1007/s00262-013-1493-8 PMC4134870 24162108

[30] Zonder JA Mohrbacher AF Singhal S van Rhee F Bensinger WI Ding H A phase 1, multicenter, open-label, dose escalation study of elotuzumab in patients with advanced multiple myeloma. Blood (2012) 120(3):552–9. 10.1182/blood-2011-06-360552 PMC4467882 22184404

[31] Lonial S Dimopoulos M Palumbo A White D Grosicki S Spicka I Elotuzumab Therapy for Relapsed or Refractory Multiple Myeloma. N Engl J Med (2015) 373 p(7):621–31. 10.1056/NEJMoa1505654 26035255

[32] Dimopoulos MA Dytfeld D Grosicki S Moreau P Takezako N Hori M Elotuzumab plus Pomalidomide and Dexamethasone for Multiple Myeloma. N Engl J Med (2018) 379(19):1811–22. 10.1056/NEJMoa1805762 30403938

[33] Lin P Owens R Tricot G Wilson CS Flow cytometric immunophenotypic analysis of 306 cases of multiple myeloma. Am J Clin Pathol (2004) 121(4):482–8. 10.1309/74R4TB90BUWH27JX 15080299

[34] Moreno L Perez C Zabaleta A Manrique I Alignani D Ajona D The Mechanism of Action of the Anti-CD38 Monoclonal Antibody Isatuximab in Multiple Myeloma. Clin Cancer Res (2019) 25(10):3176–87. 10.1158/1078-0432.CCR-18-1597 30692097

[35] van de Donk N Usmani SZ CD38 Antibodies in Multiple Myeloma: Mechanisms of Action and Modes of Resistance. Front Immunol (2018) 9:2134. 10.3389/fimmu.2018.02134 30294326 PMC6158369

[36] Sanchez L Wang Y Siegel DS Wang ML Daratumumab: a first-in-class CD38 monoclonal antibody for the treatment of multiple myeloma. J Hematol Oncol (2016) 9(1):51. 10.1186/s13045-016-0283-0 27363983 PMC4929758

[37] de Weers M Tai YT van der Veer MS Bakker JM Vink T Jacobs DC Daratumumab, a novel therapeutic human CD38 monoclonal antibody, induces killing of multiple myeloma and other hematological tumors. J Immunol (2011) 186(3):1840–8. 10.4049/jimmunol.1003032 21187443

[38] Overdijk MB Verploegen S Bogels M van Egmond M Lammerts van Bueren JJ Mutis T Antibody-mediated phagocytosis contributes to the anti-tumor activity of the therapeutic antibody daratumumab in lymphoma and multiple myeloma. MAbs (2015) 7(2):311–21. 10.1080/19420862.2015.1007813 PMC4622648 25760767

[39] Lokhorst HM Plesner T Laubach JP Nahi H Gimsing P Hansson M Targeting CD38 with Daratumumab Monotherapy in Multiple Myeloma. N Engl J Med (2015) 373(13):1207–19. 10.1056/NEJMoa1506348 26308596

[40] Lonial S Weiss BM Usmani SZ Singhal S Chari A Bahlis NJ Daratumumab monotherapy in patients with treatment-refractory multiple myeloma (SIRIUS): an open-label, randomised, phase 2 trial. Lancet (2016) 387(10027):1551–60. 10.1016/S0140-6736(15)01120-4 26778538

[41] Dimopoulos MA Oriol A Nahi H San-Miguel J Bahlis NJ Usmani SZ Daratumumab, Lenalidomide, and Dexamethasone for Multiple Myeloma. N Engl J Med (2016) 375(14):1319–31. 10.1056/NEJMoa1607751 27705267

[42] Facon T Kumar S Plesner T Orlowski RZ Moreau P Bahlis N Daratumumab plus Lenalidomide and Dexamethasone for Untreated Myeloma. N Engl J Med (2019) 380(22):2104–15. 10.1056/NEJMoa1817249 PMC10045721 31141632

[43] Palumbo A Chanan-Khan A Weisel K Nooka AK Masszi T Beksac M Daratumumab, Bortezomib, and Dexamethasone for Multiple Myeloma. N Engl J Med (2016) 375(8):754–66. 10.1056/NEJMoa1606038 27557302

[44] Chari A Suvannasankha A Fay JW Arnulf B Kaufman JL Ifthikharuddin JJ Daratumumab plus pomalidomide and dexamethasone in relapsed and/or refractory multiple myeloma. Blood (2017) 130(8):974–81. 10.1182/blood-2017-05-785246 PMC5570682 28637662

[45] Mateos MV Dimopoulos MA Cavo M Suzuki K Jakubowiak A Knop S Daratumumab plus Bortezomib, Melphalan, and Prednisone for Untreated Myeloma. N Engl J Med (2018) 378(6):518–28. 10.1056/NEJMoa1714678 29231133

[46] Moreau P Attal M Hulin C Arnulf B Belhadj K Benboubker L Bortezomib, thalidomide, and dexamethasone with or without daratumumab before and after autologous stem-cell transplantation for newly diagnosed multiple myeloma (CASSIOPEIA): a randomised, open-label, phase 3 study. Lancet (2019) 394(10192):29–38. 10.1016/S0140-6736(19)31240-1 31171419

[47] Mateos MV Nahi H Legiec W Grosicki S Vorobyev V Spicka I Subcutaneous versus intravenous daratumumab in patients with relapsed or refractory multiple myeloma (COLUMBA): a multicentre, open-label, non-inferiority, randomised, phase 3 trial. Lancet Haematol (2020) 7(5):e370–80. 10.1016/S2352-3026(20)30070-3 32213342

[48] Jiang H Acharya C An G Zhong M Feng X Wang L SAR650984 directly induces multiple myeloma cell death via lysosomal-associated and apoptotic pathways, which is further enhanced by pomalidomide. Leukemia (2016) 30(2):399–408. 10.1038/leu.2015.240 26338273

[49] Deckert J Wetzel MC Bartle LM Skaletskaya A Goldmacher VS Vallee F SAR650984, a novel humanized CD38-targeting antibody, demonstrates potent antitumor activity in models of multiple myeloma and other CD38+ hematologic malignancies. Clin Cancer Res (2014) 20(17):4574–83. 10.1158/1078-0432.CCR-14-0695 24987056

[50] Krejcik J Casneuf T Nijhof IS Verbist B Bald J Plesner T Daratumumab depletes CD38+ immune regulatory cells, promotes T-cell expansion, and skews T-cell repertoire in multiple myeloma. Blood (2016) 128(3):384–94. 10.1182/blood-2015-12-687749 PMC4957162 27222480

[51] Feng X Zhang L Acharya C An G Wen K Qiu L Targeting CD38 Suppresses Induction and Function of T Regulatory Cells to Mitigate Immunosuppression in Multiple Myeloma. Clin Cancer Res (2017) 23(15):4290–300. 10.1158/1078-0432.CCR-16-3192 PMC5540790 28249894

[52] Attal M Richardson PG Rajkumar SV San-Miguel J Beksac M Spicka I Isatuximab plus pomalidomide and low-dose dexamethasone versus pomalidomide and low-dose dexamethasone in patients with relapsed and refractory multiple myeloma (ICARIA-MM): a randomised, multicentre, open-label, phase 3 study. Lancet (2019) 394(10214):2096–107. 10.1016/S0140-6736(19)32556-5 31735560

[53] McCombs JR Owen SC Antibody drug conjugates: design and selection of linker, payload and conjugation chemistry. AAPS J (2015) 17(2):339–51. 10.1208/s12248-014-9710-8 PMC4365093 25604608

[54] Cho SF Anderson KC Tai YT Targeting B Cell Maturation Antigen (BCMA) in Multiple Myeloma: Potential Uses of BCMA-Based Immunotherapy. Front Immunol (2018) 9:1821. 10.3389/fimmu.2018.01821 30147690 PMC6095983

[55] Xing L Lin L Yu T Li Y Cho SF Liu J A novel BCMA PBD-ADC with ATM/ATR/WEE1 inhibitors or bortezomib induce synergistic lethality in multiple myeloma. Leukemia (2020) 34(8):2150–62. 10.1038/s41375-020-0745-9 PMC7392808 32060401

[56] Trudel S Lendvai N Popat R Voorhees PM Reeves B Libby EN Targeting B-cell maturation antigen with GSK2857916 antibody-drug conjugate in relapsed or refractory multiple myeloma (BMA117159): a dose escalation and expansion phase 1 trial. Lancet Oncol (2018) 19(12):1641–53. 10.1016/S1470-2045(18)30576-X PMC6328058 30442502

[57] Kinneer K Meekin J Tiberghien AC Tai YT Phipps S Kiefer CM SLC46A3 as a Potential Predictive Biomarker for Antibody-Drug Conjugates Bearing Noncleavable Linked Maytansinoid and Pyrrolobenzodiazepine Warheads. Clin Cancer Res (2018) 24(24):6570–82. 10.1158/1078-0432.CCR-18-1300 30131388

[58] Tai YT Mayes PA Acharya C Zhong MY Cea M Cagnetta A Novel anti-B-cell maturation antigen antibody-drug conjugate (GSK2857916) selectively induces killing of multiple myeloma. Blood (2014) 123(20):3128–38. 10.1182/blood-2013-10-535088 PMC4023420 24569262

[59] Trudel S Lendvai N Popat R Voorhees PM Reeves B Libby EN Antibody-drug conjugate, GSK2857916, in relapsed/refractory multiple myeloma: an update on safety and efficacy from dose expansion phase I study. Blood Cancer J (2019) 9(4):37. 10.1038/s41408-019-0196-6 30894515 PMC6426965

[60] Benson DM Jr Bakan CE Mishra A Hofmeister CC Efebera Y Becknell B The PD-1/PD-L1 axis modulates the natural killer cell versus multiple myeloma effect: a therapeutic target for CT-011, a novel monoclonal anti-PD-1 antibody. Blood (2010) 116(13):2286–94. 10.1182/blood-2010-02-271874 PMC3490105 20460501

[61] Yu B Jiang T Liu D BCMA-targeted immunotherapy for multiple myeloma. J Hematol Oncol (2020) 13(1):125. 10.1186/s13045-020-00962-7 32943087 PMC7499842

[62] Lonial S Lee HC Badros A Trudel S Nooka AK Chari A Belantamab mafodotin for relapsed or refractory multiple myeloma (DREAMM-2): a two-arm, randomised, open-label, phase 2 study. Lancet Oncol (2020) 21(2):207–21. 10.1016/S1470-2045(19)30788-0 31859245

[63] Smits NC Sentman CL Bispecific T-Cell Engagers (BiTEs) as Treatment of B-Cell Lymphoma. J Clin Oncol (2016) 34(10):1131–3. 10.1200/JCO.2015.64.9970 PMC5085271 26884583

[64] Fu M He Q Guo Z Zhou X Li H Zhao L Therapeutic Bispecific T-Cell Engager Antibody Targeting the Transferrin Receptor. Front Immunol (2019) 10:1396. 10.3389/fimmu.2019.01396 31293575 PMC6598450

[65] Seckinger A Delgado JA Moser S Moreno L Neuber B Grab A Target Expression, Generation, Preclinical Activity, and Pharmacokinetics of the BCMA-T Cell Bispecific Antibody EM801 for Multiple Myeloma Treatment. Cancer Cell (2017) 31(3):396–410. 10.1016/j.ccell.2017.02.002 28262554

[66] Hipp S Tai YT Blanset D Deegen P Wahl J Thomas O A novel BCMA/CD3 bispecific T-cell engager for the treatment of multiple myeloma induces selective lysis in vitro and in vivo. Leukemia (2017) 31(8):1743–51. 10.1038/leu.2016.388 28025583

[67] Zou J Chen D Zong Y Ye S Tang J Meng H Immunotherapy based on bispecific T-cell engager with hIgG1 Fc sequence as a new therapeutic strategy in multiple myeloma. Cancer Sci (2015) 106(5):512–21. 10.1111/cas.12631 PMC4452151 25664501

[68] Lejeune M Kose MC Duray E Einsele H Beguin Y Caers J Bispecific, T-Cell-Recruiting Antibodies in B-Cell Malignancies. Front Immunol (2020) 11:762. 10.3389/fimmu.2020.00762 32457743 PMC7221185

[69] Topp MS Duell J Zugmaier G Attal M Moreau P Langer C Anti-B-Cell Maturation Antigen BiTE Molecule AMG 420 Induces Responses in Multiple Myeloma. J Clin Oncol (2020) 38(8):775–83. 10.1200/JCO.19.02657 31895611

[70] Cho SF Lin L Xing L Li Y Wen K Yu T The immunomodulatory drugs lenalidomide and pomalidomide enhance the potency of AMG 701 in multiple myeloma preclinical models. Blood Adv (2020) 4(17):4195–207. 10.1182/bloodadvances.2020002524 PMC7479960 32898244

[71] Morgan RA Dudley ME Wunderlich JR Hughes MS Yang JC Sherry RM Cancer regression in patients after transfer of genetically engineered lymphocytes. Science (2006) 314(5796):126–9. 10.1126/science.1129003 PMC2267026 16946036

[72] Rosenberg SA Packard BS Aebersold PM Solomon D Topalian SL Toy ST Use of tumor-infiltrating lymphocytes and interleukin-2 in the immunotherapy of patients with metastatic melanoma. A Prelim Rep N Engl J Med (1988) 319(25):1676–80. 10.1056/NEJM198812223192527 3264384

[73] Rosenberg SA Restifo NP Adoptive cell transfer as personalized immunotherapy for human cancer. Science (2015) 348(6230):62–8. 10.1126/science.aaa4967 PMC6295668 25838374

[74] Noonan KA Huff CA Davis J Lemas MV Fiorino S Bitzan J Adoptive transfer of activated marrow-infiltrating lymphocytes induces measurable antitumor immunity in the bone marrow in multiple myeloma. Sci Transl Med (2015) 7(288):288ra78. 10.1126/scitranslmed.aaa7014 PMC4634889 25995224

[75] Rapoport AP Stadtmauer EA Binder-Scholl GK Goloubeva O Vogl DT Lacey SF NY-ESO-1-specific TCR-engineered T cells mediate sustained antigen-specific antitumor effects in myeloma. Nat Med (2015) 21(8):914–21. 10.1038/nm.3910 PMC4529359 26193344

[76] Thomas R Al-Khadairi G Roelands J Hendrickx W Dermime S Bedognetti D NY-ESO-1 Based Immunotherapy of Cancer: Current Perspectives. Front Immunol (2018) 9:947. 10.3389/fimmu.2018.00947 29770138 PMC5941317

[77] D’Agostino M Raje N Anti-BCMA CAR T-cell therapy in multiple myeloma: can we do better? Leukemia (2020) 34(1):21–34. 10.1038/s41375-019-0669-4 31780814

[78] Eshhar Z Waks T Gross G Schindler DG Specific activation and targeting of cytotoxic lymphocytes through chimeric single chains consisting of antibody-binding domains and the gamma or zeta subunits of the immunoglobulin and T-cell receptors. Proc Natl Acad Sci U.S.A. (1993) 90(2):720–4. 10.1073/pnas.90.2.720 PMC45737 8421711

[79] Garcia-Guerrero E Sierro-Martinez B Perez-Simon JA Overcoming Chimeric Antigen Receptor (CAR) Modified T-Cell Therapy Limitations in Multiple Myeloma. Front Immunol (2020) 11:1128. 10.3389/fimmu.2020.01128 32582204 PMC7290012

[80] Lee DW Gardner R Porter DL Louis CU Ahmed N Jensen M Current concepts in the diagnosis and management of cytokine release syndrome. Blood (2014) 124(2):188–95. 10.1182/blood-2014-05-552729 PMC4093680 24876563

[81] Barrett DM Teachey DT Grupp SA Toxicity management for patients receiving novel T-cell engaging therapies. Curr Opin Pediatr (2014) 26(1):43–9. 10.1097/MOP.0000000000000043 PMC4198063 24362408

[82] Ali SA Shi V Maric I Wang M Stroncek DF Rose JJ T cells expressing an anti-B-cell maturation antigen chimeric antigen receptor cause remissions of multiple myeloma. Blood (2016) 128(13):1688–700. 10.1182/blood-2016-04-711903 PMC5043125 27412889

[83] Garfall AL Maus MV Hwang WT Lacey SF Mahnke YD Melenhorst JJ Chimeric Antigen Receptor T Cells against CD19 for Multiple Myeloma. N Engl J Med (2015) 373(11):1040–7. 10.1056/NEJMoa1504542 PMC4646711 26352815

[84] Raje N Berdeja J Lin Y Siegel D Jagannath S Madduri D Anti-BCMA CAR T-Cell Therapy bb2121 in Relapsed or Refractory Multiple Myeloma. N Engl J Med (2019) 380(18):1726–37. 10.1056/NEJMoa1817226 PMC8202968 31042825

[85] Jelinek T Mihalyova J Kascak M Duras J Hajek R PD-1/PD-L1 inhibitors in haematological malignancies: update 2017. Immunology (2017) 152(3):357–71. 10.1111/imm.12788 PMC5629439 28685821

[86] Ishida Y Agata Y Shibahara K Honjo T Induced expression of PD-1, a novel member of the immunoglobulin gene superfamily, upon programmed cell death. EMBO J (1992) 11(11):3887–95. 10.1002/j.1460-2075.1992.tb05481.x PMC556898 1396582

[87] Zou W Chen L Inhibitory B7-family molecules in the tumour microenvironment. Nat Rev Immunol (2008) 8(6):467–77. 10.1038/nri2326 18500231

[88] Gorgun G Samur MK Cowens KB Paula S Bianchi G Anderson JE Lenalidomide Enhances Immune Checkpoint Blockade-Induced Immune Response in Multiple Myeloma. Clin Cancer Res (2015) 21(20):4607–18. 10.1158/1078-0432.CCR-15-0200 PMC4609232 25979485

[89] Hallett WH Jing W Drobyski WR Johnson BD Immunosuppressive effects of multiple myeloma are overcome by PD-L1 blockade. Biol Blood Marrow Transplant (2011) 17(8):1133–45. 10.1016/j.bbmt.2011.03.011 21536144

[90] Paiva B Azpilikueta A Puig N Ocio EM Sharma R Oyajobi BO PD-L1/PD-1 presence in the tumor microenvironment and activity of PD-1 blockade in multiple myeloma. Leukemia (2015) 29(10):2110–3. 10.1038/leu.2015.79 25778100

[91] Liu J Hamrouni A Wolowiec D Coiteux V Kuliczkowski K Hetuin D Plasma cells from multiple myeloma patients express B7-H1 (PD-L1) and increase expression after stimulation with IFN-{gamma} and TLR ligands via a MyD88-, TRAF6-, and MEK-dependent pathway. Blood (2007) 110(1):296–304. 10.1182/blood-2006-10-051482 17363736

[92] Tamura H Ishibashi M Yamashita T Tanosaki S Okuyama N Kondo A Marrow stromal cells induce B7-H1 expression on myeloma cells, generating aggressive characteristics in multiple myeloma. Leukemia (2013) 27(2):464–72. 10.1038/leu.2012.213 22828443

[93] Ray A Das DS Song Y Richardson P Munshi NC Chauhan D Targeting PD1-PDL1 immune checkpoint in plasmacytoid dendritic cell interactions with T cells, natural killer cells and multiple myeloma cells. Leukemia (2015) 29(6):1441–4. 10.1038/leu.2015.11 PMC5703039 25634684

[94] Tamura H Ishibashi M Sunakawa-Kii M Inokuchi K PD-L1-PD-1 Pathway in the Pathophysiology of Multiple Myeloma. Cancers (Basel) (2020) 12(4):924. 10.3390/cancers12040924 PMC7226506 32290052

[95] Lesokhin AM Ansell SM Armand P Scott EC Halwani A Gutierrez M Nivolumab in Patients With Relapsed or Refractory Hematologic Malignancy: Preliminary Results of a Phase Ib Study. J Clin Oncol (2016) 34(23):2698–704. 10.1200/JCO.2015.65.9789 PMC5019749 27269947

[96] Ribrag V Avigan DE Green DJ Wise-Draper T Posada JG Vij R Phase 1b trial of pembrolizumab monotherapy for relapsed/refractory multiple myeloma: KEYNOTE-013. Br J Haematol (2019) 186(3):e41–4. 10.1111/bjh.15888 30937889

[97] Costello C The future of checkpoint inhibition in multiple myeloma? Lancet Haematol (2019) 6(9):e439–40. 10.1016/S2352-3026(19)30149-8 31327688

[98] Postow MA Sidlow R Hellmann MD Immune-Related Adverse Events Associated with Immune Checkpoint Blockade. N Engl J Med (2018) 378(2):158–68. 10.1056/NEJMra1703481 29320654

[99] Jelinek T Paiva B Hajek R Update on PD-1/PD-L1 Inhibitors in Multiple Myeloma. Front Immunol (2018) 9:2431. 10.3389/fimmu.2018.02431 30505301 PMC6250817

[100] Oliva S Troia R D’Agostino M Boccadoro M Gay F Promises and Pitfalls in the Use of PD-1/PD-L1 Inhibitors in Multiple Myeloma. Front Immunol (2018) 9:2749. 10.3389/fimmu.2018.02749 30538704 PMC6277686

[101] Costa F Das R Kini Bailur J Dhodapkar K Dhodapkar MV Checkpoint Inhibition in Myeloma: Opportunities and Challenges. Front Immunol (2018) 9:2204. 10.3389/fimmu.2018.02204 30319648 PMC6168958

[102] Guo C Manjili MH Subjeck JR Sarkar D Fisher PB Wang XY Therapeutic cancer vaccines: past, present, and future. Adv Cancer Res (2013) 119:421–75. 10.1016/B978-0-12-407190-2.00007-1 PMC3721379 23870514

[103] Maurer DM Butterfield LH Vujanovic L Melanoma vaccines: clinical status and immune endpoints. Melanoma Res (2019) 29(2):109–18. 10.1097/CMR.0000000000000535 PMC6392068 30802228

[104] Garfall AL Stadtmauer EA Cellular and vaccine immunotherapy for multiple myeloma. Hematol Am Soc Hematol Educ Program (2016) 2016(1):521–7. 10.1182/asheducation-2016.1.521 PMC6142464 27913524

[105] Cohen AD Raje N Fowler JA Mezzi K Scott EC Dhodapkar MV How to Train Your T Cells: Overcoming Immune Dysfunction in Multiple Myeloma. Clin Cancer Res (2020) 26(7):1541–54. 10.1158/1078-0432.CCR-19-2111 PMC8176627 31672768

[106] Le DT Pardoll DM Jaffee EM Cellular vaccine approaches. Cancer J (2010) 16(4):304–10. 10.1097/PPO.0b013e3181eb33d7 PMC3086689 20693840

[107] Dranoff G GM-CSF-based cancer vaccines. Immunol Rev (2002) 188:147–54. 10.1034/j.1600-065X.2002.18813.x 12445288

[108] Rapoport AP Aqui NA Stadtmauer EA Vogl DT Xu YY Kalos M Combination immunotherapy after ASCT for multiple myeloma using MAGE-A3/Poly-ICLC immunizations followed by adoptive transfer of vaccine-primed and costimulated autologous T cells. Clin Cancer Res (2014) 20(5):1355–65. 10.1158/1078-0432.CCR-13-2817 PMC4557204 24520093

[109] Tsuboi A Oka Y Nakajima H Fukuda Y Elisseeva OA Yoshihara S Wilms tumor gene WT1 peptide-based immunotherapy induced a minimal response in a patient with advanced therapy-resistant multiple myeloma. Int J Hematol (2007) 86(5):414–7. 10.1007/BF02983998 18192109

[110] Bae J Smith R Daley J Mimura N Tai YT Anderson KC Myeloma-specific multiple peptides able to generate cytotoxic T lymphocytes: a potential therapeutic application in multiple myeloma and other plasma cell disorders. Clin Cancer Res (2012) 18(17):4850–60. 10.1158/1078-0432.CCR-11-2776 PMC3839582 22753586

[111] Bae J Prabhala R Voskertchian A Brown A Maguire C Richardson P A multiepitope of XBP1, CD138 and CS1 peptides induces myeloma-specific cytotoxic T lymphocytes in T cells of smoldering myeloma patients. Leukemia (2015) 29(1):218–29. 10.1038/leu.2014.159 PMC4237716 24935722

[112] Bae J Samur M Richardson P Munshi NC Anderson KC Selective targeting of multiple myeloma by B cell maturation antigen (BCMA)-specific central memory CD8(+) cytotoxic T lymphocytes: immunotherapeutic application in vaccination and adoptive immunotherapy. Leukemia (2019) 33(9):2208–26. 10.1038/s41375-019-0414-z PMC6728221 30872779

[113] Hansson L Abdalla AO Moshfegh A Choudhury A Rabbani H Nilsson B Long-term idiotype vaccination combined with interleukin-12 (IL-12), or IL-12 and granulocyte macrophage colony-stimulating factor, in early-stage multiple myeloma patients. Clin Cancer Res (2007) 13(5):1503–10. 10.1158/1078-0432.CCR-06-1603 17332295

[114] McCann KJ Godeseth R Chudley L Mander A Di Genova G Lloyd-Evans P Idiotypic DNA vaccination for the treatment of multiple myeloma: safety and immunogenicity in a phase I clinical study. Cancer Immunol Immunother (2015) 64(8):1021–32. 10.1007/s00262-015-1703-7 PMC4506484 25982371

[115] Coscia M Mariani S Battaglio S Di Bello C Fiore F Foglietta M Long-term follow-up of idiotype vaccination in human myeloma as a maintenance therapy after high-dose chemotherapy. Leukemia (2004) 18(1):139–45. 10.1038/sj.leu.2403181 14574332

[116] Rosenblatt J Vasir B Uhl L Blotta S Macnamara C Somaiya P Vaccination with dendritic cell/tumor fusion cells results in cellular and humoral antitumor immune responses in patients with multiple myeloma. Blood (2011) 117(2):393–402. 10.1182/blood-2010-04-277137 21030562 PMC3031474

[117] Rosenblatt J Avivi I Vasir B Uhl L Munshi NC Katz T Vaccination with dendritic cell/tumor fusions following autologous stem cell transplant induces immunologic and clinical responses in multiple myeloma patients. Clin Cancer Res (2013) 19(13):3640–8. 10.1158/1078-0432.CCR-13-0282 PMC3755905 23685836

[118] Romano E Cotari JW Barreira da Silva R Betts BC Chung DJ Avogadri F Human Langerhans cells use an IL-15R-alpha/IL-15/pSTAT5-dependent mechanism to break T-cell tolerance against the self-differentiation tumor antigen WT1. Blood (2012) 119(22):5182–90. 10.1182/blood-2011-09-382200 PMC3369609 22510877

[119] Chung DJ Romano E Pronschinske KB Shyer JA Mennecozzi M St Angelo ET Langerhans-type and monocyte-derived human dendritic cells have different susceptibilities to mRNA electroporation with distinct effects on maturation and activation: implications for immunogenicity in dendritic cell-based immunotherapy. J Transl Med (2013) 11:166. 10.1186/1479-5876-11-166 23837662 PMC3710267

[120] Benboubker L Dimopoulos MA Dispenzieri A Catalano J Belch AR Cavo M Lenalidomide and dexamethasone in transplant-ineligible patients with myeloma. N Engl J Med (2014) 371(10):906–17. 10.1056/NEJMoa1402551 25184863

[121] Durie BGM Hoering A Abidi MH Rajkumar SV Epstein J Kahanic SP Bortezomib with lenalidomide and dexamethasone versus lenalidomide and dexamethasone alone in patients with newly diagnosed myeloma without intent for immediate autologous stem-cell transplant (SWOG S0777): a randomised, open-label, phase 3 trial. Lancet (2017) 389(10068):519–27. 10.1016/S0140-6736(16)31594-X PMC5546834 28017406

[122] O’Donnell EK Laubach JP Yee AJ Chen T Huff CA Basile FG A phase 2 study of modified lenalidomide, bortezomib and dexamethasone in transplant-ineligible multiple myeloma. Br J Haematol (2018) 182(2):222–30. 10.1111/bjh.15261 PMC6074026 29740809

[123] Joseph NS Kaufman JL Dhodapkar MV Hofmeister CC Almaula DK Heffner LT Long-Term Follow-Up Results of Lenalidomide, Bortezomib, and Dexamethasone Induction Therapy and Risk-Adapted Maintenance Approach in Newly Diagnosed Multiple Myeloma. J Clin Oncol (2020) 38(17):1928–37. 10.1200/JCO.19.02515 PMC7587409 32298201

[124] Moreau P Masszi T Grzasko N Bahlis NJ Hansson M Pour L Oral Ixazomib, Lenalidomide, and Dexamethasone for Multiple Myeloma. N Engl J Med (2016) 374(17):1621–34. 10.1056/NEJMoa1516282 27119237

[125] Stewart AK Rajkumar SV Dimopoulos MA Masszi T Spicka I Oriol A Carfilzomib, lenalidomide, and dexamethasone for relapsed multiple myeloma. N Engl J Med (2015) 372(2):142–52. 10.1056/NEJMoa1411321 25482145

[126] Richardson PG Oriol A Beksac M Liberati AM Galli M Schjesvold F Pomalidomide, bortezomib, and dexamethasone for patients with relapsed or refractory multiple myeloma previously treated with lenalidomide (OPTIMISMM): a randomised, open-label, phase 3 trial. Lancet Oncol (2019) 20(6):781–94. 10.1016/S1470-2045(19)30152-4 31097405

[127] Kronke J Udeshi ND Narla A Grauman P Hurst SN McConkey M Lenalidomide causes selective degradation of IKZF1 and IKZF3 in multiple myeloma cells. Science (2014) 343(6168):301–5. 10.1126/science.1244851 PMC4077049 24292625

[128] Lu G Middleton RE Sun H Naniong M Ott CJ Mitsiades CS The myeloma drug lenalidomide promotes the cereblon-dependent destruction of Ikaros proteins. Science (2014) 343(6168):305–9. 10.1126/science.1244917 PMC4070318 24292623

[129] Gandhi AK Kang J Havens CG Conklin T Ning Y Wu L Immunomodulatory agents lenalidomide and pomalidomide co-stimulate T cells by inducing degradation of T cell repressors Ikaros and Aiolos via modulation of the E3 ubiquitin ligase complex CRL4(CRBN.). Br J Haematol (2014) 164(6):811–21. 10.1111/bjh.12708 PMC4232904 24328678

[130] Hideshima T Ogiya D Liu J Harada T Kurata K Bae J Immunomodulatory drugs activate NK cells via both Zap-70 and cereblon-dependent pathways. Leukemia (2020) 35(1):177–88. 10.1038/s41375-020-0809-x PMC7529681 32238854

[131] Ocio EM Fernandez-Lazaro D San-Segundo L Lopez-Corral L Corchete LA Gutierrez NC In vivo murine model of acquired resistance in myeloma reveals differential mechanisms for lenalidomide and pomalidomide in combination with dexamethasone. Leukemia (2015) 29(3):705–14. 10.1038/leu.2014.238 25102946

[132] Sehgal K Das R Zhang L Verma R Deng Y Kocoglu M Clinical and pharmacodynamic analysis of pomalidomide dosing strategies in myeloma: impact of immune activation and cereblon targets. Blood (2015) 125(26):4042–51. 10.1182/blood-2014-11-611426 PMC4481593 25869284

[133] Bjorklund CC Kang J Amatangelo M Polonskaia A Katz M Chiu H Iberdomide (CC-220) is a potent cereblon E3 ligase modulator with antitumor and immunostimulatory activities in lenalidomide- and pomalidomide-resistant multiple myeloma cells with dysregulated CRBN. Leukemia (2020) 34(4):1197–201. 10.1038/s41375-019-0620-8 PMC7214241 31719682

[134] Hansen JD Correa M Nagy MA Alexander M Plantevin V Grant V Discovery of CRBN E3 Ligase Modulator CC-92480 for the Treatment of Relapsed and Refractory Multiple Myeloma. J Med Chem (2020) 63(13):6648–76. 10.1021/acs.jmedchem.9b01928 32130004

[135] Bianchi G Oliva L Cascio P Pengo N Fontana F Cerruti F The proteasome load versus capacity balance determines apoptotic sensitivity of multiple myeloma cells to proteasome inhibition. Blood (2009) 113(13):3040–9. 10.1182/blood-2008-08-172734 19164601

[136] Gandolfi S Laubach JP Hideshima T Chauhan D Anderson KC Richardson PG The proteasome and proteasome inhibitors in multiple myeloma. Cancer Metastasis Rev (2017) 36(4):561–84. 10.1007/s10555-017-9707-8 29196868

[137] Ettari R Zappala M Grasso S Musolino C Innao V Allegra A Immunoproteasome-selective and non-selective inhibitors: A promising approach for the treatment of multiple myeloma. Pharmacol Ther (2018) 182:176–92. 10.1016/j.pharmthera.2017.09.001 28911826

[138] Spisek R Charalambous A Mazumder A Vesole DH Jagannath S Dhodapkar MV Bortezomib enhances dendritic cell (DC)-mediated induction of immunity to human myeloma via exposure of cell surface heat shock protein 90 on dying tumor cells: therapeutic implications. Blood (2007) 109(11):4839–45. 10.1182/blood-2006-10-054221 PMC1885516 17299090

[139] Hull EE Montgomery MR Leyva KJ HDAC Inhibitors as Epigenetic Regulators of the Immune System: Impacts on Cancer Therapy and Inflammatory Diseases. BioMed Res Int (2016) 2016:8797206. 10.1155/2016/8797206 27556043 PMC4983322

[140] Bae J Hideshima T Tai YT Song Y Richardson P Raje N Histone deacetylase (HDAC) inhibitor ACY241 enhances anti-tumor activities of antigen-specific central memory cytotoxic T lymphocytes against multiple myeloma and solid tumors. Leukemia (2018) 32(9):1932–47. 10.1038/s41375-018-0062-8 PMC6537609 29487385

[141] Laubach JP van de Donk N Davies FE Mikhael J Practical Considerations for Antibodies in Myeloma. Am Soc Clin Oncol Educ Book (2018) 38:667–74. 10.1200/EDBK_205443 30231321

[142] Laurent SA Hoffmann FS Kuhn PH Cheng Q Chu Y Schmidt-Supprian M gamma-Secretase directly sheds the survival receptor BCMA from plasma cells. Nat Commun (2015) 6:7333. 10.1038/ncomms8333 26065893 PMC4490565

[143] Pont MJ Hill T Cole GO Abbott JJ Kelliher J Salter AI gamma-Secretase inhibition increases efficacy of BCMA-specific chimeric antigen receptor T cells in multiple myeloma. Blood (2019) 134(19):1585–97. 10.1182/blood.2019000050 PMC6871311 31558469

